# Partnership delivery of evidence-based therapy intervention to improve upper-limb function: a retrospective analysis

**DOI:** 10.1136/bmjpo-2025-003572

**Published:** 2025-07-16

**Authors:** Jill Massey, Leanne Dreyer, Erika Molteni, Ben Siegle, Tomoki Arichi, Anne Gordon

**Affiliations:** 1 Evelina London Children’s Hospital Neurosciences Department, London, UK; 2 Florence Nightingale School of Nursing Midwifery and Palliative Care, King’s College London, London, UK; 3 Institute of Health Informatics, University College London, London, UK; 4 Department of Perinatal Imaging & Health, King’s College London, London, UK

**Keywords:** Rehabilitation, Occupational therapy, Cerebral Palsy, Health Policy, Infant

## Abstract

**Objective:**

To describe clinical characteristics and outcomes of children and young people with hemiplegia completing a novel dedicated therapy intervention, ‘Evelina REACH’. This evidence-based upper-limb outpatient intervention is delivered in partnership with the child, caregivers, and occupational therapists at a tertiary hospital and community allied health professionals.

**Design:**

Retrospective audit of patients completing a 6-week protocolised therapy intervention with repeated standardised measures of spontaneous arm and hand use and of caregiver goal rating.

**Setting:**

A tertiary level children’s hospital in London, UK.

**Patients:**

156 children (median age 26 months, range 4 months–16.5 years) completing a therapy intervention programme between 2012 and 2023.

**Interventions:**

An intensive, protocolised and individualised goal-directed therapy intervention programme, co-delivered by the patient, caregivers and hospital-based and community therapists.

**Main outcome measures:**

Assisting Hand Assessment (AHA), Goal Attainment Scaling (GAS)/Goal Attainment Scaling Light and Canadian Occupational Performance Measure (COPM).

**Results:**

Clinically significant gains were achieved with a mean AHA logit score change of 7 (n=69) which was maintained at 6 weeks post intervention (n=35). At least 1 GAS goal was met or exceeded by 99.2% participants, with measurable score change across three caregiver-mediated COPM goals, performance=90.17%, and satisfaction=83.58%. Of caregivers surveyed, 97.85% would repeat the programme, and 100% would recommend it to others.

**Conclusions:**

Evelina REACH is a clinically effective, goal-directed intensive activity-based therapy intervention that fosters lasting functional improvements in upper-limb use. Further research should explore optimal and scalable co-delivery models to enhance children’s access to evidence-based therapy in statutory healthcare.

**WHAT IS ALREADY KNOWN ON THIS TOPIC**

- Specific upper-limb therapy interventions can lead to lasting change in hand function for children with hemiplegia, yet such models are rarely available in routine healthcare.

**WHAT THIS STUDY ADDS**

- Clinically effective evidence-based upper-limb therapy intervention can be delivered within statutory health services using a partnership model involving children, caregivers and therapists.
- The intervention results in quantifiable change in spontaneous bimanual hand use in daily life activities, sustained beyond intervention completion.

**HOW THIS STUDY MIGHT AFFECT RESEARCH, PRACTICE OR POLICY**

- A protocolised intervention approach provides the opportunity to evidence the effectiveness of therapy intervention.
- Partnership models of intervention delivery may enhance equitable delivery of evidence-based upper-limb therapy by facilitating knowledge and skill sharing between therapists.
- Intensive goal-directed upper-limb therapy demands substantial resources but provides long-term benefits. Service commissioning adjustments may improve access to these interventions.

## Introduction

 Neuromotor disorders encompass a range of conditions affecting motor control and coordination. Among these, cerebral palsy (CP) is the most common cause of physical disability in childhood.^1^ Hemiplegic CP, characterised by unilateral motor impairment, accounts for 38% of CP cases, primarily affecting motor control on one side of the body.^2^ This asymmetry often impacts children’s ability to participate in everyday activities, influencing their quality of life and independence.^1^ For the purposes of this paper, the term ‘children’ encompasses infants, children and young people, while ‘caregivers’ includes parents and carers.

Robust evidence indicates intensive time-bound, individualised goal-directed therapy (GDT) interventions that facilitate repetitive functional child-initiated movement can produce lasting improvements in hand and arm function for children with hemiplegic CP.^3^ These evidence-based intervention modalities; constraint-induced movement therapy (CIMT), bimanual therapy (BT) and GDT,^4^ have primarliy been studied with school-aged children. Increasingly, evidence shows early intervention effectively improves outcomes in children with hemiplegia.^5^ Grounded in motor learning theory, these therapies emphasise the type of task, type of practice and type of feedback,^6^,^8^ with therapists tailoring delivery to individual and contextual factors.

There are widely recognised barriers to the translation of evidence into paediatric therapy practice at health service organisation and practitioner levels.^9^,^11^ This is relevant, as while randomised control trials show that high-dose, intensive interventions are effective, they are resource-heavy and access within the UK National Health Service (NHS) remains limited.

Evelina REACH is a tertiary, occupational therapist-led service, based at Evelina London Children’s Hospital (ELCH), a regional UK children’s hospital. Evelina REACH provides intensive, goal-directed, evidence-based therapy intervention for children (6 months−16 years), with unilateral motor deficits, regardless of aetiology or timing of injury. Intervention is delivered in partnership with children, caregivers, the child’s community-based therapist (occupational or physiotherapist) and ELCH occupational therapists. The intervention aims to achieve lasting functional improvement in spontaneous use of the child’s affected arm and hand. Intervention programmes are protocolised, with individualised goals tailored to the child’s age (dosage and intensity), clinical presentation (modality) and family and child preference.

The aims of this paper are to describe: (1) the ‘Evelina REACH’ model of partnership upper-limb intervention, (2) the clinical characteristics and outcomes of 156 children who completed a 6-week Evelina REACH programme, (3) the relationship between goal achievement (Goal Attainment Scaling (GAS)) and satisfaction (Canadian Occupational Performance Measure (COPM)) scores with changes in upper limb function (Assisting Hand Assessment (AHA)/Mini-AHA) in response to intervention.

## Method

### Referral criteria

Evelina REACH referral criteria include children aged 6 months–16 years, with clinical signs of hemiplegia or asymmetric four limb CP, who can actively grasp and release with one hand at enrolment, and where the child and caregivers are motivated to improve hand or arm use. Referrals originate from primary care, hospital-based or community-based medical professionals for consultation and advice or intensive intervention consideration. Children are not eligible if: (1) they have fixed contractures or deformity, (2) they have a diagnosed neurodegenerative condition or (3) intensive intervention is considered unlikely to be tolerated or desired due to individual or family self-reported priorities or lifestyle factors.

### Clinical protocol

**Figure 1:**
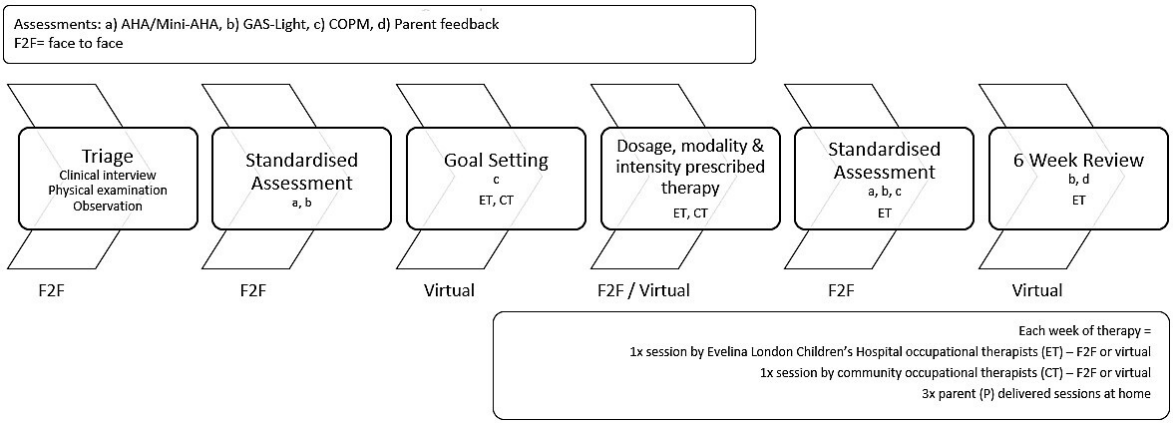
Evelina REACH programme pathway. AHA, Assisting Hand Assessment; COPM, Canadian Occupational Performance Measure; GAS-Light, Goal Attainment Scaling Light.

Intervention modalities provided are BT, a hybrid of BT and modified CIMT (mCIMT) or GDT, typically delivered over 6 weeks. Protocols (modality and dosage) are guided by the child’s age, cognitive, motor, attentional and adaptive behaviours (based on interview, standardised and observational assessment findings) and child and caregiver goals. Individual intervention combines face-to-face and virtual sessions, in partnership between children, caregivers, community-based therapists and the Evelina REACH team. Children have in-person assessments and goal setting with ELCH therapists at the beginning and end of intervention. During the intervention, children typically see ELCH and a community therapist weekly for 1 hour each (in person or virtually), with caregivers delivering the remaining dosage at home (figure 1). Further ad hoc liaison between sessions takes place as required. Dosage and intensity are prescribed in minutes and days per week, age-banded (table 1) based on best available evidence and expert consensus^12^ and recorded in a patient-held logbook by all delivery partners.

**Table 1:** Age group and distributed dosage

Age banding	Distributed dosage—6-week period
Infants (3–12 months)	30 min per day; 5–6 days/week Total: 15–18 hours
Preschoolers (1 year–4.5 years)	45 min – 1.5 hours per day; 5–6 days/week Total: 22.5–54 hours
School-aged children	2 hours per day; 5–6 days/week Total: 60–72 hours

Prior to intervention, motor function is classified using the Gross Motor Function Classification System (GMFCS) Expanded and Revised^13^ and, where appropriate, the Manual Ability Classification System (MACS)^14^ or Mini-MACS (Mini-MACS).^15^

### Patient and public involvement

The service follows continuous improvement principles, with regular input from children, families and therapists through engagement activities and a reference group. While this is a retrospective audit of clinical data, the original service design—and thus the research questions and outcome measures—were shaped by caregiver priorities. Caregivers and children were not involved in the audit’s conduct or recruitment, but results will be shared with families and stakeholders through established communication channels.

### Standardised assessments

The Vineland Adaptive Behavior Scale version II^16^ or version III^17^ Parent/Caregiver Questionnaire is completed prior to intervention to obtain a measure of functional daily life abilities and to support goal setting. Standard scores are calculated for overall adaptive behaviour, and for communication, socialisation and daily living.

To evaluate intervention impact on activity and participation, standardised age-specific assessments are administered immediately before, immediately after and 6 weeks post-intervention (figure 1). The AHA suite of measures assesses spontaneous use of both hands during a semistructured play session: the Mini-AHA for children 8–18 months^18^ and the Kids AHA for children 18 months–18 years (referred to as AHA). ^19^ Written consent is obtained for video assessments for later scoring by a trained assessor. Raw scores are converted to logit scores for evaluation. As Mini-AHA and AHA logit scores are not commensurate, they are analysed separately. A logit score change of >5 in the AHA is considered clinically significant.^20^ The COVID-19 pandemic and associated staffing restrictions impacted the consistency of Mini-AHA and AHA administration across the study population for a period of time.

The GAS^21^ or Goal Attainment Scaling Light (GAS-Light)^22^ is administered and scored by an ELCH occupational therapist through observation of the child engaging in up to three preferred bimanual play activities. The COPM^23^ is administered as a semistructured interview with the caregivers and/or child to identify up to three priority daily activities, for example, putting on shoes and opening a lunch box. Likert scale scoring of 1–10 for performance and satisfaction is recorded.

During BT programmes, GAS and COPM goals inform and evaluate the intervention and translation of skills to daily activities, including those not directly practised during the intervention. In contrast, in GDT, GAS and COPM goals inform the intervention content with a whole task practice approach.^4^

An additional AHA was initially completed 6 weeks preintervention to capture spontaneous improvements or local therapy-related changes in hand and arm function. This time point was later removed from the protocol to improve clinical efficiency and reduce burden, and findings from this subset are reported in this paper.

At the end of the intervention, partnering caregivers and community therapists provide feedback through semistructured interview or online survey. Three key questions are asked of families. (1) Was the effort of doing the programme worth the outcome? (2) Would you consider doing it again? (3) Would you recommend it to others? Community therapists are asked if they would be willing to partner again in intervention delivery.

The Strengthening the Reporting of Observational Studies in Epidemiology Checklist was used to ensure transparent and robust reporting.^24^

### Analysis

Therapy intervention outcomes and clinical change over time were characterised in terms of Mini-AHA/AHA logit scores, change in Likert score (COPM) or GAS (0=goal achieved, 1=goal exceeded).

Wilcoxon rank test was used to test for significant standardised score changes between protocol time points with a significant p value defined as <0.05. Distribution of score changes at each time point was calculated according to quartiles. Spearman’s rho explored correlations between goal achievement (GAS) and caregiver performance and satisfaction (COPM) scores with changes in upper limb function (AHA/Mini-AHA).

## Results

Findings are reported for a consecutive sample of 156/163 children, triaged for intervention. Seven were excluded from analysis: three followed a different protocol, and four did not complete the recommended intervention programme due to factors including child’s tolerance and family circumstances.

### Patient population

From April 2012 to December 2023, 156 children aged 4 months–16.5 years completed the intervention programme. Over two-thirds of the patients resided in Greater London (table 2). 60% of referrals originated from ELCH doctors and 40% from external doctors.

**Table 2:** Patient demographics (n=156)

Age at commencement of programme	Median: 26 months, IQR (16–44)
Age range	
Preschool (<48 months of age):	117 (75.0%)
Primary school (48–131 months):	31 (19.9%)
Secondary school (132 months or older):	6 (3.8%)
Gender	86 male (55.1%)
	70 female (44.9%)
More affected side (n=154)	Left=57 (37.0%)
	Right=97 (63.0%)
Gross Motor Function Classification Scale (GMFCS) (n=137)	GMFCS 1: 68 (49.6%)
	GMFCS 2: 43 (31.4%)
	GMFCS 3: 17 (12.4%)
	GMFCS 4: 9 (6.6%)
	GMFCS 5: 0 (0.0%)
Manual Ability Classification Scale (MACS) / Mini-MACS (n=133)	All Mini-MACS: 97
	Mini-MACS 1: 6 (6.2%)
	Mini-MACS 2: 24 (24.7%)
	Mini-MACS 3: 53 (54.6%)
	Mini-MACS 4: 12 (12.4%)
	Mini-MACS 5: 2 (2.1%)
	All MACS: 36
	MACS 1: 2 (5.6%)
	MACS 2: 20 (55.6%)
	MACS 3: 10 (27.8%)
	MACS 4: 4 (11.1%)
	MACS 5: 0 (0.0%)
Ethnicity (n=156)	White British: 50 (32.0%)
	Not stated/specified: 97 (62.2%)
	Other: 9 (5.8%)
Place of residence	Greater London: 112 (72%)
	Counties across the UK: 44 (28%)
Quintile of deprivation (n=153)	Quintile 1: 8 (5.2%)
	Quintile 2: 40 (26.1%)
	Quintile 3: 38 (24.8%)
	Quintile 4: 36 (23.5%)
	Quintile 5: 31 (20.3%)

iqr, interquartile range.

Three quarters of the children were preschool aged (<48 months old). The majority of children had a GMFCS of level 1–3 (93.4%) and Mini-MACS or MACS score of 1–3 (88.9%).

The English Indices of Multiple Deprivation 2019^25^ were used to provide a proxy measure of relative socioeconomic deprivation for each child. Although all deprivation quintiles are represented in our study population (table 2), the distribution of deprivation in this sample is less deprived than the typical population in London.^26^ Patient ethnicity was classified using the NHS data dictionary^27^ and extracted from health records where availability was limited.

The children’s adaptive behaviour abilities prior to intervention (online supplemental table S1) indicated around half of those participating scored more than 1 SD below the population mean. Daily living and functional motor skills were the two most impaired domains of function.

### Intervention

BT was the most common intervention modality delivered, typically involving two caregivers in the partnership. All sessions were undertaken in person until April 2020, when the service transitioned to a hybrid face-to-face and virtual model (via secure video conferencing) in response to the COVID-19 pandemic. Of the 156 patients, 81 received hybrid programmes (table 3).

**Table 3:** Intervention programmes delivered

n=156	
Prescribed intervention modality	
Hybrid (mCIMT+BT):	20 (12.8%)
mCIMT:	10 (6.4%)
BT:	94 (60.3%)
GDT	32 (20.5%)
Partners (n=156)	
Both caregivers	82 (52.6%)
Mother	69 (44.2%)
Father	4 (2.6%)
Aunt/uncle	1 (0.6%)
Delivery method	
In person	61 (39.1%)
Virtual	14 (9.0%)
Hybrid	81 (51.9%)
% of set dosage achieved (n=122)	
Mean	0.976
SD	0.205
Median	0.97
IQR	(0.9;1.438)

BT, bimanual therapy; GDT, goal directed therapy; IQR, interquartile range; mCIMT, modified constraint induced movement therapy; SD, standard deviation.

The prescribed dosage for an individual intervention programme varied from 10 hours to 60 hours (median 22.5 hours). Dosage records were available for 122/156 participants, with dosage achieved ranging from 7.5 hours to 68.75 hours (median 19.8 hours). A total of 60 participants (49.18%) achieved or exceeded the target dose (table 3).

### Clinical outcomes

The findings of the intervention across the key time points up to 3 months post intervention are outlined in online supplemental table S2.

#### Assisting Hand Assessment

Preintervention and postintervention AHA or Mini-AHA scores were available for 69/156 patients. A median score change of 7 AHA logits was found between preintervention and postintervention assessments (Mini-AHA IQR=(4–11); AHA IQR=(5–10.5)) (online supplemental table S2). However, a Wilcoxon rank test indicated that there was no significant difference between either the mini-AHA score at the beginning of intervention (mean rank=−1.93, p=0.05) and at end of intervention (mean rank=−1.59, p=0.11) or the AHA at beginning of intervention (mean rank=−0.03, p=0.97) and at end of intervention (mean rank=−1.41, p=0.16).

#### Goal attainment

GAS goals were recorded for 133/156 patients, 67 GAS and 71 GAS-Light (table 4). Nearly all patients (132/133) achieved an expected score change (or greater) in one or more goals, with a clinically significant score change across three caregiver-mediated COPM goals, performance=90.17% and satisfaction=83.58% (table 4).

**Table 4:** GAS And COPM score changes at end of treatment

GAS achievements at the end of treatment			
n=133	At least one goal	At least two goals	At least three goals
Score >1	132	124	95
Score >0	94	54	20
Score > −1	20	7	2

COPM Achievements at the end of treatment			
n=138	At least one goal	At least two goals	At least three goals
Score ≥2 satisfaction	134	131	118
Score ≥2 performance	134	132	117

COPM, Canadian Occupational Performance Measure; GAS, Goal Attainment Scaling.

#### Relationship between dimensions

**Figure 2:**
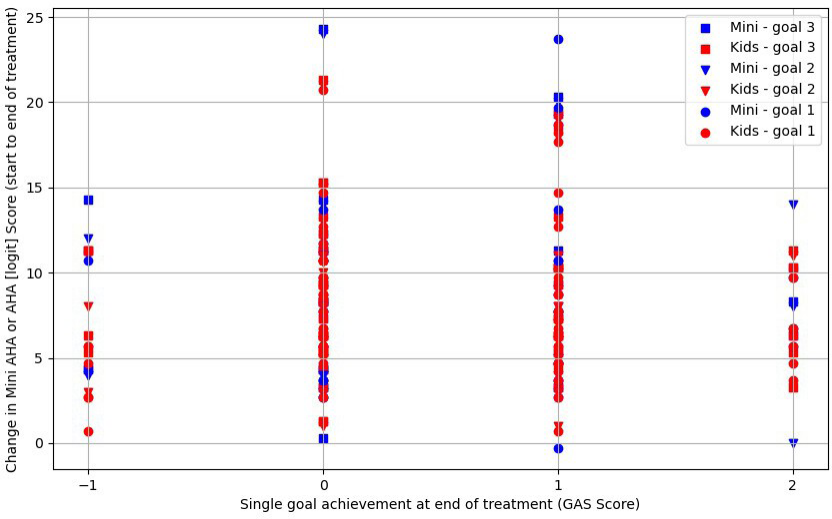
Goal achievement versus change in upper limb function (Mini-AHA/AHA scores), (n=84). AHA, Assisting Hand Assessment; GAS, Goal Attainment Scaling.

**Figure 3:**
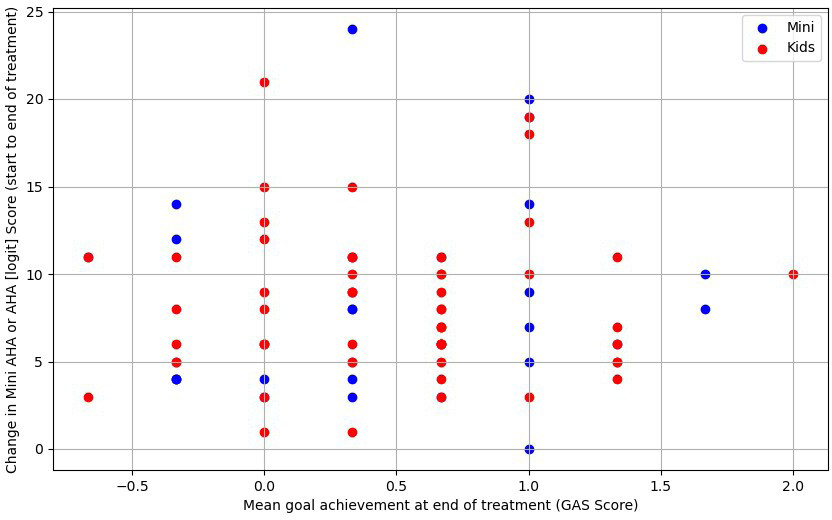
Mean GAS goal achievement versus change in upper limb function (Mini-AHA/AHA score), (n=84). AHA, Assisting Hand Assessment; GAS, Goal Attainment Scaling.

**Figure 4:**
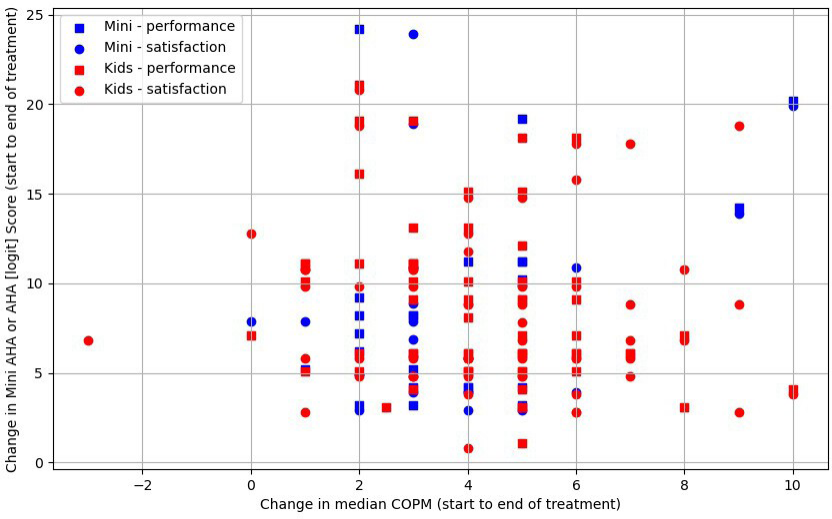
Caregiver performance and satisfaction score changes (COPM) and change in upper limb function (Mini-AHA/Kids AHA) outcomes, n=83. AHA, Assisting Hand Assessment; COPM, Canadian Occupational Performance Measure.

Figure 2 demonstrates no clear relationship between GAS goal attainment (up to three goals per child) and changes in AHA or Mini-AHA logic scores. Based on mean GAS goal outcomes per child, figure 3 shows no apparent association with AHA/Mini-AHA score changes. Figure 4 also suggests no relationship between changes in logit scores and median COPM outcomes (performance and satisfaction). In summary, no significant correlations were found, aside from one likely false positive (for further detail, see online supplemental tables S3–S5).

End of intervention score changes were not related to the presenting MACS or mini-MACS score, indicating in this cohort the level of baseline function did not relate to AHA evaluated score changes (online supplemental figure S1).

#### Caregiver and partnering community therapist feedback

At intervention completion between March 2022 and April 2024, 124 caregivers received an online survey. A total of 98 caregivers responded, and 93 completed all questions. Prior to the use of online surveys, 13 caregivers took part in structured end of intervention interviews. All caregivers across the surveys or interviews reported the effort was worth the outcome, they would recommend the programme to others and 97.85% would undertake the programme again. At intervention completion, 108 partnering community therapists received an online survey, 41 responded and 38 completed all questions. All partnering community therapists (100%) reported that they would be willing to partner again to deliver the intervention.

#### 6-week repeated baseline assessment prior to intervention

In the subgroup of 30 children who received repeated standardised assessments 6 weeks before and immediately prior to intervention, no change in median AHA or Mini-AHA logit scores was found. There was no significant difference between children that had preintervention repeated assessments compared with the remainder of the cohort in baseline AHA/Mini-AHA logit scores.

## Discussion

We describe successful delivery of a model of intensive, protocolised upper-limb intervention which is individualised and goal-directed. This model can be effectively delivered in partnership between children, caregivers, hospital and community therapists. These findings align with existing research showing that structured upper-limb interventions are both safe and clinically effective.^3 5 28 29^ Protocolised upper-limb intervention enables the routine data capture of clinical outcomes, using standardised assessments and goal attainment.

The lack of significant change in median AHA logit scores for the initial sub-group of children assessed 6 weeks prior to commencing intervention suggests that any improvements are unlikely to be related to intrinsic age-related improvement or routine therapy provision. These findings align with previous research indicating that goal-directed activity-based high-intensity intensive upper-limb interventions can achieve greater functional gains and improved bimanual coordination for children with hemiplegia compared with lower dose standard care.^30^,^32^

The partnership model of delivering evidence-based upper-limb intervention with children, caregivers, hospital and community therapists was tolerated and accepted by children and their caregivers. 70% of caregivers responded reporting high satisfaction, regardless of clinical outcomes, and 35% of partnering community therapists expressed willingness to partner again and valued the shared delivery model. A recent article^33^ on the experiences of a subgroup of participants described the process as demanding but manageable and supportive of knowledge development.

Dosage achieved in this study aligns with reported ranges and varies by intervention modality.^34^ The high achievement of dose reported may reflect participant motivation and the protocol including therapist time and materials to support education of caregivers and children around intervention selection and demands and allowing time to consider impact on family before the decision to proceed. Dosage prescription is guided by emerging evidence and clinical reasoning.

Supporting community-based therapists, particularly those in isolated roles with diverse caseloads, is crucial for maintaining fidelity and effective delivery. It has been described that paediatric intervention fidelity is influenced by variability in therapist training, experience, familiarity and adherence to motor learning principles.^35 36^ Regular targeted training for therapists is critical to effective and responsive individualised intervention delivery and optimal clinical outcomes. Variability in engagement, motivation and clinical presentation necessitates flexibility to maintain fidelity while ensuring sufficient intensity and dosage.^8^ Evelina REACH conducts informal workshops to engage community-based colleagues, reinforce expectations, develop supportive networks and share evidence-based knowledge.

Delivering this intervention via a hospital-based regional centre ensured provision of a standardised approach to delivery across a region with wide variation in local therapy availability. A key limitation of this model, even with hybrid delivery approaches, is the difficulty of hospital-based therapists observing the child in their natural environments, such as home and school. The partnership model addresses this challenge by enabling caregivers and community-based therapists to integrate therapy into everyday routines and individual lifestyles. There is no single optimal model for delivery of such interventions in statutory healthcare; however, considerations include availability of and training for appropriately skilled clinicians to respond to local and regional demands across a health system.

As this was a retrospective audit of a clinical service, limitations such as population heterogeneity and sample size limited the ability to analyse age-related differences and moderating and mediating factors in relation to clinical outcomes. Limited ethnicity data in patient health records restricted exploration, and this requires further development in evaluating access inequities. Strengths include real-world implementation of intensive, protocolised upper-limb intervention, aligned with evidence on caregiver-delivered models. Future research should explore age-related responses, particularly in relation to infants and preschoolers, as published evidence is more prevalent in school-aged children.

Caregiver and young person education, self-efficacy and meaningful goal setting are core components of the Evelina REACH programme. Further evaluation of models of evidence-based interventions, including from a health economics perspective, is needed to establish sustainability. Rising therapy service pressures and growing waiting lists^37^ highlight the urgent need for effective, scalable intervention models. Intensive goal-directed upper-limb therapy demands significant resource investment but offers long-term benefits, including improved functional independence and reduced future healthcare burden. Beyond motor outcomes, such interventions enhance child well-being, participation and physical health.^38^ Self-care abilities strongly correlate to bimanual performance, reinforcing the importance of targeted intervention.^39^ Additionally, these services can support professional development, extended scope roles and align with broader healthcare strategies aimed at maximising children’s potential. Innovations in digital technology, placing delivery closer to home and school and reducing dependence on hospital-based visits are indicated.

## Conclusions

Intensive, protocolised intervention programmes with individualised goals, delivered collaboratively between children, caregivers, hospital and community therapists, are feasible and effective. These interventions lead to measurable improvements in upper-limb function, achievement of activity-based goals and caregiver-reported performance and satisfaction of daily functioning. Sustained benefits at intervention end and 6-week follow-up highlight the potential of partnership models and hybrid delivery to improve access by reducing travel demands. The COVID-19 pandemic also showed that hybrid delivery may extend reach to children in remote or low-resource settings. Social, cultural, educational and digital barriers must be addressed, and family circumstances, school attendance, caregiver availability and therapist access are considered to ensure equitable implementation.

## Supplementary material

10.1136/bmjpo-2025-003572 online supplemental file 1

## Data Availability

No data are available.
