# Supplementary material for: Partnership delivery of evidence-based therapy intervention to improve upper-limb function: a retrospective analysis
Source: BMJ Paediatr Open. 2025 Jul 16;9(1):e003572. doi: 10.1136/bmjpo-2025-003572 (PMC12273171; doi:10.1136/bmjpo-2025-003572)
Supplement: online supplemental file 1 [file bmjpo-9-1-s001.docx]

**Supplementary Material**

**Table S1. Pre-intervention (baseline) adaptive Behaviour Skills; Vineland II and Vineland III subdomain scores.**

| **Vineland II score** | **N** | **Adaptive Behaviour Composite** | **Communication** | **Daily Living** | **Socialisation** | **Motor** |
| --- | --- | --- | --- | --- | --- | --- |
| Median  Overall:  Pre-school:  School: | 18  13  5 | 84.5  87.0  84.0 | 93.0  93.0  97.0 | 87.0  91.0  87.0 | 93.0  92.0  96.0 | 76.5  80.0  67.0 |
| IQR  Overall:  Pre-school:  School: | 18  13  5 | [79.5; 91.3]  [79.0; 98.0]  [82.0; 85.0] | [85.5; 99.8]  [85.0; 99.0]  [89.0; 100.0] | [81.3; 98.8]  [85.0; 100.0]  [77.0; 87.0] | [86.0; 100.8]  [86.0; 101.0]  [86.0; 100.0] | [68.8; 81.0]  [74.0; 87.0]  [64.0; 75.0] |
| N (%) scoring below 85    Overall:  Pre-school:  School: | 18  13  5 | 9 (50.0)%  6 (46.2%)  3 (60.0%) | 4 (22.2%)  3 (23.1%)  1 (20.0%) | 5 (27.8%)  3 (23.1%)  2 (40.0%) | 3 (16.7%)  2 (15.4%)  1 (20.0%) | 14 (77.8%)  9 (69.2%)  5 (100.0%) |
| **Vineland III score** | **N** | **Adaptive Behaviour Composite** | **Communication** | **Daily Living** | **Socialisation** | **Motor** |
| Median  Overall:  Pre-school:  School: | 110  88  20 | 84.0  85.0  79.0 | 88.0  89.0  86.5 | 84.0  85.0  79.5 | 88.0  88.0  88.0 | 76.0  76.0  71.0 |
| IQR  Overall:  Pre-school:  School: | 110  88  20 | [76.0; 93.0]  [77.0; 93.0]  [72.0; 99.5] | [79.25; 95.0]  [79.75; 95.0]  [78.25; 101.25] | [73.0; 90.0]  [78.0; 91.0]  [66.0; 85.5] | [81.0; 98.0]  [83.0; 98.0]  [76.5; 98.0] | [65.0; 84.75]  [66.0; 85.0]  [62.0; 77.0] |
| Mean (Std. Dev.)  Overall:  Pre-school:  School: | 110  88  20 | 84.3 (13.8)  84.9 (12.6)  82.7 (18.4) | 86.2 (16.9)  86.4 (15.1)  86.7 (23.5) | 81.5 (17.3)  83.1 (16.3)  75.0 (20.2) | 89.5 (13.8)  90.2 (12.3)  87.6 (19.0) | 72.7 (19.4)  73.8 (19.6)  68.2 (18.1) |
| N (%) scoring below 85  Overall:  Pre-school:  School: | 110  88  20 | 56 (50.9%)  42 (47.7%)  12 (60.0%) | 43 (39.1%)  32 (36.4%)  9 (45.0%) | 56 (50.9%)  40 (45.5%)  14 (70.0%) | 38 (34.5%)  27 (30.7%)  9 (45.0%) | 76 (69.1%)  63 (71.6%)  11 (55.0%) |

**Figure S1. Mini-AHA/ AHA vs. Mini MACS/ MACS (n=82)**
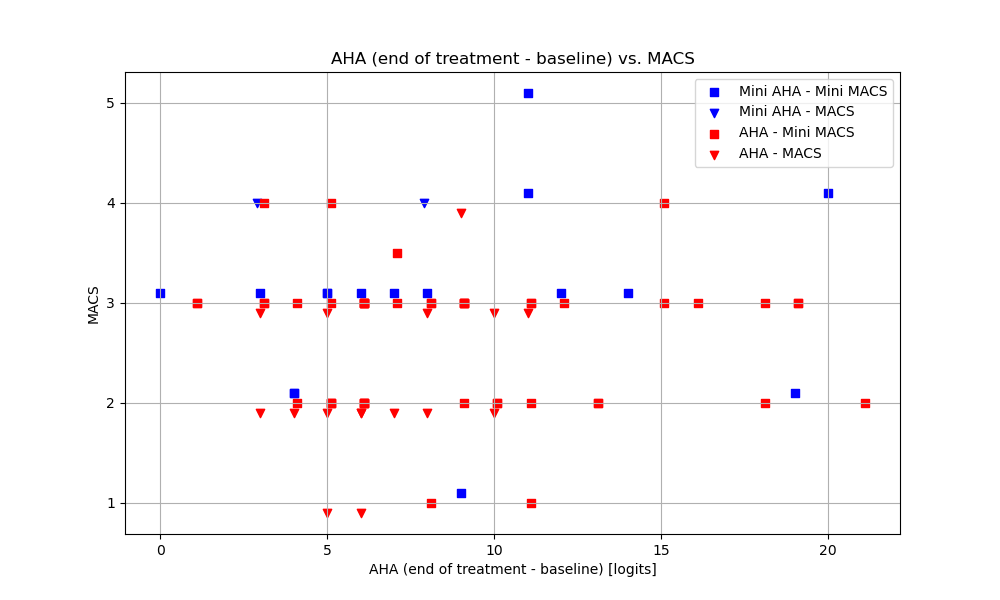


Change in Mini AHA/ AHA [logit] start to end of treatment

Mini MAS/ MACS

**Table S2: Clinical Outcomes Standardised Assessments At Beginning And End Of Treatment, And At 3-Month Follow Up.**

| **Assessment** | **N T0** | **T0 Score** | **N T1** | **T1**  **Score** | **N T2** | **T2**  **Score** | **N T3** | **T3**  **Score** | **N T4** | **T4**  **Score** | **N T5** | **ΔT0–T1** | **ΔT2–T1** | **ΔT3–T1** | **ΔT4–T1** |
| --- | --- | --- | --- | --- | --- | --- | --- | --- | --- | --- | --- | --- | --- | --- | --- |
| **Mini-AHA (logits)** | 6 | 23.5 [11.75; 27.75] | 29 | 23 [16; 46] | 28 | 31 [23.75; 51] | 8 | 27.0 [21.5; 40.0] | 3 | 25 [20.5; 39.0] | 2 | 18.5 [11.25; 25.75] | 7.5 [4;11] | 11.0 [5.0; 20.0] | 0  [-2; 4.5] |
| **AHA (logits)** | 26 | 45.0  [35.0; 49.0] | 78 | 45 [28; 52.75 | 69 | 53 [33;61] | 35 | 52.0 [39.5; 57.0] | 7 | 43 [36.5; 47] | 28 | 50.0 [30.75; 58.75] | 7  [5;  10.5] | 7.0 [6.0; 9.0] | 12 [2.25; 16.5] |
| **COPM performance** |  |  | 140 | 2 [1;3] | 137 | 6 [5; 7] |  |  |  |  |  |  | 4 [3; 5] |  |  |
| **COPM satisfaction** |  |  | 140 | 2 [1;4] | 137 | 7 [5; 8] |  |  |  |  |  |  | 4 [3; 6] |  |  |
| **GAS** |  |  | 138 | n.a defined as –1 for each participant | 132 | 0.0 [0.0; 1.0] |  |  |  |  |  |  |  |  |  |

Legend: **N Tx**= Number of subjects assessed at timepoint; **Tx Score**: Median score [IQR] at timepoint Tx; **T0**: Pre-assessment median [IQR]; **T1**: Initial assessment; **T2**: End of treatment; **T3**: 6-week follow-up; **T4**: 3-month follow-up; **T5**: 6-month follow-up; **ΔTn–T1**: Median change in score from T1 to Tn [IQR] T0 = 6-weeks prior to intervention; T1 = initial assessment, immediately prior to intervention commencing; T2 = assessment at end of 6-week intervention programme; T3 = 6-week repeat assessment findings; T4 = 3 month review

**Table S3 – Correlations relating to Figure2**

|  | Goal 1 | Goal 2 | Goal 3 | Combined goals (all pooled) |
| --- | --- | --- | --- | --- |
| Spearman correlations for all | rho = 0.072  p = 0.507  N = 88 | rho = 0.104  p = 0.336  N = 88 | rho = 0.078  p = 0.469  N = 88 | rho = 0.084  p = 0.173  N = 264 |
| Spearman correlations for Mini AHA: | rho = 0.129  p = 0.531  N = 26 | rho = 0.092  p = 0.655  N = 26 | rho = 0.119  p = 0.563  N = 26 | rho = 0.108  p = 0.348  N = 78 |
| Spearman correlations for Kids AHA: | rho = -0.002, p = 0.988, N = 58 | rho = 0.071  p = 0.596  N = 58 | rho = -0.049p = 0.714,  N = 58 | rho = -0.004  p = 0.958  N = 174 |

**Table S4 Correlations relating to Figure 3**

| Spearman correlations between mean GAS goals and the difference between baseline AHA and AHA at end of treatment | |
| --- | --- |
| AHA Mini | rho = 0.122  p = 0.5537 |
| AHA Kids | rho = 0.004  p = 0.9759 |

**Table S5 Correlations relating to Figure 4**

| Spearman correlations between caregiver performance and satisfaction score changes (COPM) and change in upper limb function (mini AHA/AHA) | |
| --- | --- |
| Mini - Performance: | rho = 0.296  p = 0.1704 |
| Mini - Satisfaction: | rho = 0.120  p = 0.5857 |
| Kids - Performance: | rho = -0.292  p = 0.0235 |
| Kids - Satisfaction | rho = -0.119  p = 0.3634 |
