# Supplementary material for: Partnership delivery of evidence-based therapy intervention to improve upper-limb function: a retrospective analysis
Source: BMJ Paediatr Open. 2025 Jul 16;9(1):e003572. doi: 10.1136/bmjpo-2025-003572 (PMC12273171; doi:10.1136/bmjpo-2025-003572)
Supplement: Supplementary file 2 [file bmjpo-2025-003572.reviewer_comments.pdf]

PEER REVIEW HISTORY

BMJ Paediatrics Open publishes all reviews undertaken for accepted manuscripts. Reviewers are asked to complete a checklist review form and are provided with free text boxes to elaborate on their assessment. These free text comments are reproduced below.

ARTICLE DETAILS

**TITLE (PROVISIONAL)**	Partnership delivery of evidence-based therapy intervention to improve upper-limb function: A retrospective analysis
**AUTHORS**	Massey, Jill; Dreyer, Leanne; Molteni, Erika; Siegle, Ben; Arichi, Tomoki; Gordon, Anne

VERSION 1 - REVIEW

**REVIEWER NAME**	***Leanne Sakzewski***
**REVIEWER AFFILIATION**	University of Queensland, Queensland Cerebral Palsy and Rehabilitation Research Centre, Faculty of Medicine
**REVIEWER CONFLICT OF INTEREST**	N/A
**AI DISCLOSURE**	No
**DATE REVIEW RETURNED**	28-May-2025

**GENERAL COMMENTS**	This retrospective audit characterised the clinical characteristics and outcomes of children with unilateral cerebral palsy who received evidence-based upper limb therapy in partnership with hospital, family and community therapy providers.<br>This is a well written paper.<br>The main aspect that needs further refinement is the section regarding the "Relationship between dimensions". This is not explicitly stated as an aim of this study and needs to be included as such, along with greater details around analysis. The figures associated with this are confusing. It is unclear how a relationship between variables was determined? There only appears to be a series of figures (2-4 and S1). Were correlations performed as would be assumed when reporting on relationships between variables? I would suggest carrying out a series of correlations or other relevant statistic and reporting the values. This would be more informative than the figures which are difficult to interpret.<br>Table S1: There appears to be some misalignment of data in rows.

**REVIEWER NAME**	***Jennifer McCahill***
**REVIEWER AFFILIATION**	None disclosed
**REVIEWER CONFLICT OF INTEREST**	N/A
**AI DISCLOSURE**	No
**DATE REVIEW RETURNED**	03-Jun-2025

**GENERAL COMMENTS**	This study is a retrospective review of a novel upper limb service led by a tertiary hospital (Evelina) partnering with parents and community therapists to deliver evidence-based upper limb therapy in children with hemiplegia over 6 weeks. The children are assessed prior to intervention, immediately post intervention, and at 6 weeks

post intervention using the AHA, GAS and COPM as the primary outcome measures. Treatment varied for the 156 children with majority receiving bimanual therapy, hybrid modified CIMT and bimanual, and goal-directed therapy.

Overall the paper is very well written and an important piece of work to publish. I do have some thoughts to put to the authors, as well as clarifications I believe will benefit the paper.

Introduction
- it is suggested that 80% of children with CP have hemiplegia using a NICE reference- I believe this number to be smaller (40%)?

Methodology
- In the clinic protocol the paper states the dosage and intensity were based on 'best available evidence and expert consensus' (ref 11)- but I think the paper would benefit if the reader had more clarity on the 'protocolised' intervention decision making if possible- so this study/ protocol could be replicated by another service(s). Could this possibly be grouped into age brackets- or is it based on MACS scores? The authors also mention that the type of intervention was determined in conversation with the family- but more transparency on this would be useful for the service model to be replicated elsewhere.

Results
- In the patient population section the authors note that 75% of their population was less than 4 years old. It would be interesting to understand if this group differed in any way to the older group. The younger group still has a developing corticospinal tract and sensorimotor system with more abundant neuroplasticity where as the older group this is limited. Therefore differentiating these 2 groups is of interest. I do appreciate of course that the numbers would be less for the older group- but this could be an important consideration for future studies?

- In the intervention section the text reports 95 receiving hybrid sessions- but the table reports 81 'mixed'- the numbers should be consistent and it might be better for the table to read 'hybrid' as the text.

- The prescribed dosage was 10-60 hours- this is a great variance so the reader would benefit from understanding why the dosage varied so much (possibly with information about what type of child (age/ MACS) was prescribed 10 versus 60 hours). It is also stated here that only 49.18% achieved this dosage, but looking at the encouraging results further down (despite 49% achieving the prescribed dosage)- it seems the authors should make comment on this in the discussion that potentially their algorithm of determining dosage might need reflecting on if less achieves positive results? Or possibly a comparison of the children who received the 'correct dosage' versus those who did not?

-It is encouraging that the MACS levels did not correlate with improved logit scores indicating that severity of initial functioning did not impact the ability to improve.

- The parent feedback is impressive with 98 caregivers responding - but this should be noted that it is less than 70% feedback rate. In addition the community therapy response rate was only 38/108 -

	with 100% of those therapists willing to partner again- however this bias should be noted in the discussion section.<br><br>In conclusion, I would like to thank the authors for sharing this important service evaluation, however I believe more detail on the protocol for dosage and intensity is needed and the decision making process on how it was decided which therapy the children receive would be beneficial to the paper. As the paper is currently under word-count, hopefully the information can be included.

VERSION 1 – AUTHOR RESPONSE

12.	Reviewer 1	Relationship between dimensions	The main aspect that needs further refinement is the section regarding the "Relationship between dimensions". This is not explicitly stated as an aim of this study and needs to be included as such, along with greater details around analysis. The figures associated with this are confusing. It is unclear how a relationship between variables was determined? There only appears to be a series of figures (2-4 and S1). Were correlations performed as would be assumed when reporting on relationships between variables? I would suggest carrying out a series of correlations or other relevant statistic and reporting the values. This would be more informative than the figures which are difficult to interpret.	Pg 5 - A study aim has been included in relation to the section ‘relationship between dimensions’. Further detail has been added to the analysis section. Spearman correlations were calculated and have now been added to Supplementary Information (Table S3-S5) for transparency. The reporting of results ‘relationship between dimensions’ has also been revised.<br><br>Chart titles and axis labels have been revised to ease interpretation of the figures.
13.			Table S1: There appears to be some misalignment of data in rows	The table has been revised to rectify the misalignment.
14.	Reviewer 2	Introduction	it is suggested that 80% of children with CP have hemiplegia using a NICE reference- I believe this number to be smaller (40%)?	Pg 4. We agree, the prevalence has been amended to 39% and referenced.
15.		Methodology	- In the clinic protocol the paper states the dosage and intensity were based on 'best available evidence and expert consensus' (ref 11)- but I think the paper would benefit if the reader had more clarity on the 'protocolised' intervention decision making if possible- so this study/ protocol could be replicated by another service(s). Could this possibly be grouped into age brackets- or is it based on MACS scores?	Pg 6. We appreciate the suggestion to clarify decision-making for the protocolised intervention. While dosage followed published protocols, therapy selection was guided by clinical reasoning—based on the child’s trajectory, cognition, developmental stage, attention, and co-morbidities such as sensory impairments—rather than solely on age or MACS level. Adaptations, including visual supports, ensured accessibility. Further detail has been added including a reference which

				provides a framework for decision making, reflecting real-world clinical considerations beyond standard RCT protocols.
16.			The authors also mention that the type of intervention was determined in conversation with the family- but more transparency on this would be useful for the service model to be replicated elsewhere.	Pg 6 - The description of the clinical protocol (p. 6) has been revised to include details of child and caregiver involvement. To further clarify the process of intervention selection, we have also added information outlining the factors that guided our clinical decision-
17.		Results	- In the patient population section the authors note that 75% of their population was less than 4 years old. It would be interesting to understand if this group differed in any way to the older group. The younger group still has a developing corticospinal tract and sensorimotor system with more abundant neuroplasticity where as the older group this is limited. Therefore differentiating these 2 groups is of interest. I do appreciate of course that the numbers would be less for the older group- but this could be an important consideration for future studies?	Pg 13/14 - We agree that neurodevelopmental differences between younger and older children—particularly in relation to corticospinal tract maturation and neuroplasticity—are a consideration. However, exploring differences between these age groups is beyond the scope of the current study, particularly given the small number of older participants. We have, however, added a note in the discussion on pg 13/14 highlighting the need for further research to explore potential differences in intervention response between preschool and school-aged children. We also highlight that the current evidence base is more robust for older children, constraint-induced movement therapy and bimanual therapies have demonstrated efficacy in older children and emerging evidence is available for their application to infants younger than 2 years of age.
18.			- In the intervention section the text reports 95 receiving hybrid sessions- but the table reports 81 'mixed'- the numbers should be consistent and it might be better for the table to read 'hybrid' as the text.	P9 & pg 23 - This has been updated for consistency between the text and table
19.			- The prescribed dosage was 10-60 hours- this is a great variance so the reader would benefit from understanding why the dosage varied so much (possibly with information about what type of child (age/ MACS) was prescribed 10 versus 60 hours). It is also stated here that only 49.18% achieved this dosage, but looking at the encouraging results further down (despite 49% achieving the prescribed	Pg 12 We have addressed this in the discussion, noting that the wide dosage range reflects individualised clinical reasoning.

			dosage)- it seems the authors should make comment on this in the discussion that potentially their algorithm of determining dosage might need reflecting on if less achieves positive results? Or possibly a comparison of the children who received the 'correct dosage' versus those who did not?	
20.			-It is encouraging that the MACS levels did not correlate with improved logit scores indicating that severity of initial functioning did not impact the ability to improve.	Thank you for your comment. The size of this sample did not allow us to explore impact of wider factors in addition to MACS.
21.			- The parent feedback is impressive with 98 caregivers responding - but this should be noted that it is less than 70% feedback rate. In addition the community therapy response rate was only 38/108 - with 100% of those therapists willing to partner again- however this bias should be noted in the discussion section.	Pg 12 – thank you this has been noted in the discussion section
22.			In conclusion, I would like to thank the authors for sharing this important service evaluation, however I believe more detail on the protocol for dosage and intensity is needed and the decision making process on how it was decided which therapy the children receive would be beneficial to the paper. As the paper is currently under word-count, hopefully the information can be included.	We believe we have now addressed this and clarified the protocol and decision making through response to the helpful comments from the reviewers
